# Interobserver reliability and diagnostic accuracy of prehospital triage for identifying traumatic brain injury in paediatric patients: a systematic review

**DOI:** 10.1007/s00381-023-06144-3

**Published:** 2023-10-18

**Authors:** Sara Alsuwais, Naif Alqurashi, Richard Body, Simon Carley

**Affiliations:** 1 Division of Cardiovascular Sciences, University of Manchester, Manchester, UK; 2 Department of Emergency Medical Services, College of Applied Medical Sciences, King Saud bin Abdul-Aziz University for Health Sciences, Riyadh, Saudi Arabia; 3 Emergency Department, Manchester University NHS Foundation Trust, Manchester, UK

## Abstract

**Purpose:**

The consistency and accuracy of paediatric TBI triage tools can be affected by different factors, such as patients’ characteristics and the level of knowledge and skill of the caregiver. This systematic review included all the available data on the level of agreement between paramedics and ED physicians about the reliability of tools to identify paediatric TBI and the diagnostic accuracy of several such tools in prehospital settings when used by paramedics.

**Methods:**

MEDLINE (OVID), EMBASE (OVID), Cochrane Library (OVID), and CINAHL Plus (EBSCO) databases were searched from inception to 27 October 2022. Quality, bias, and applicability were assessed using COSMIN for interobserver reliability studies and QUADAS-2 tool for diagnostic accuracy studies. Narrative synthesis was employed because data were unsuitable for meta-analysis.

**Results:**

Initial searches identified 660 papers in total. Five met the inclusion criteria. Two studies showed moderate agreement between paramedics and ED physicians for GCS assessment. The PTS overtriage rate was 10% and the undertriage rate was 62%, while the triage tape had an overtriage rate of 18% and an undertriage rate of 68%. Pre-hospital GCS had 86.67% sensitivity and 71.43% specificity [95% CI]: 0.74–0.96 for neurosurgically significant TBI.

**Conclusion:**

Low level of GCS agreement and poor diagnostic accuracy may cause further harm to the patient; thus, further studies are recommended to improve the prehospital management of children with head injuries.

## Background

To optimise outcomes for paediatric with TBI, careful management of the condition is crucial from the moment of injury and often for many years [1]. The immediate priorities for paramedics to assist children with TBI are to ensure complete stabilisation and triage and move the injured patient to a centre with appropriate care [2]. Several studies have indicated that the impact of prehospital care on children with TBI is great, especially in terms of mitigating secondary TBI and improving the child’s neurological status [3–6]. To achieve this goal, it is essential to highlight the significant role of paramedics in triaging children with TBI accurately. Several paediatric triage tools were identified for use in prehospital and in-hospital fields. Some were devolved from tools for triaging adults and modified for paediatric use later, like Glasgow coma scale (GCS) [7]. Others, such as paediatric trauma score (PTS), were explicitly developed to triage trauma children [8]. However, triaging children with TBI may be challenging because of the anatomy and physiology of children [9–11] and the different levels of paramedics’ confidence when dealing with paediatric cases rather than adults [12]. As a result, paramedics often over-triages children with trauma injuries, so medical resources are overused [13]. According to the American College of Surgeons Committee on Trauma (ACS-COT), a minimum of 5% undertriage might be essential to eradicate any life-threatening undertriage [14]. Triage methods aim to obtain the lowest possible undertriage rate in adult and paediatric patients. This can be seen in the appropriate management and assessment and decreased mortality and morbidity [15, 16]. Some tools, such as GCS, are routinely calculated as part of the patient’s essential care [17]. The GCS is considered one of the most common tools for classifying TBI patients in prehospital and in-hospital settings. It is now routinely calculated as an essential part of the patient assessment [18–20].

The consistency and accuracy of paediatric TBI triage tools can be affected by factors such as patients’ characteristics and the caregiver’s knowledge and skills [21]. Therefore, it is crucial to evaluate the performance of these tools. Numerous systematic reviews have investigated TBI in children and their prehospital management. Guidelines for children with TBI have also been published. Nevertheless, the literature lacks an overview of the diagnostic accuracy and interobserver reliability of triage tools used for the condition in the prehospital setting. Thus, in this review, all the available data on paediatric TBI prehospital triage tools level of agreement between paramedics and ED physicians and the diagnostic accuracy of these tools when applied by paramedics.

## Methods

This systematic review was conducted according to the Preferred Reporting in Systematic Reviews and Meta-Analyses (PRISMA) guidelines [22]. The research team developed the review protocol and registered at the International Prospective Register of Systematic Reviews (PROSPERO) on 21st November 2022 (CRD42022365155).

The following databases were searched from inception to 27 October 2022: MEDLINE (OVID), EMBASE (OVID), Cochrane Library (OVID), and CINAHL Plus (EBSCO). Using Boolean operators, a stepwise search strategy was performed to identify all papers related to prehospital paediatric TBI. Then, a specific search was performed on each triage tool. To ensure all relevant literature was included, a grey literature search was conducted using Google Scholar search engine, OpenGrey, pre-prints (MedRxiv), and dissertation databases. The reference list from each included study was checked manually to find related articles. Examples of searches for the diagnostic accuracy of paediatric prehospital TBI triage tools and interobserver reliability are displayed in Table 1.

**Table 1:** Examples of search strategies for the diagnostic accuracy of paediatric TBI prehospital triage tools and interobserver reliability

***Database***	***Search Terms***		***Search terms cont***			
			*Interobserver reliability search*		*Diagnostic accuracy search*	
*MEDLINE (OVID)*	#S1	“Emergency medical services” OR EMS.mp. OR prehospital$.mp. OR pre-hospital$.mp. OR “out of hospital”.mp. OR paramedic$.mp. OR ambulance.mp	#S6	Interobserver.mp OR reliability.mp. OR agreement.mp	#S8	Accuracy.mp OR over-triage.mp. OR under-triage.mp
	*#S2*	*“Traumatic brain injury”.mp. or exp brain injuries, traumatic/OR TBI OR “head injury$”.mp. OR Concussion$.mp*	*#S7*	*S5 AND S6*	*#S9*	*S5 AND S8*
	*#S3*	*Paediatric$.mp. OR paediatric$.mp. OR child$.mp. OR kid$.mp. OR adolesce$.mp. OR teenage$.mp*				
	#S4	Triage.mp				
	#S5	S1 AND S2 AND S3 AND S4				

mp = title, book title, abstract, original title, name of substance word, subject heading word, floating sub-heading word, keyword heading word, organism supplementary concept word

All identified articles were uploaded to EndNote software to remove duplicates. The studies were then exported to the Rayyan Qatar Computing Research Institute (QCRI) web tool for systematic reviews for title and abstract screening by two reviewers (SA and NA) independently.

### Eligibility criteria and data collection

Regardless of the year of publication, full-text studies that used retrospective, prospective, and randomised control trial (RCT) methods written in English and reported on prehospital paediatric patients (aged < 16 years) diagnosed with TBI of any severity were eligible to be included. Editorials, conference papers and opinion articles, studies not reporting paediatric TBI and studies including hospital-based paediatric TBI were excluded from this review. After the relevant papers were identified by title and abstract, a comprehensive full-text reading was performed by (SA and NA) to identify the most relevant studies. A third reviewer (RB) was consulted when there was uncertainty about the eligibility of a study.

Data on the interobserver reliability and diagnostic accuracy of triage tools were extracted using an electronic data extraction form previously developed and assessed by the review team to check its accuracy and validity. SA AND NA extracted the data, including information about a study’s characteristics, such as title, aim, publication year, and patients’ characteristics, including sample size, age, and inclusion, and exclusion criteria. For interobserver reliability studies, data on the agreement test and the level of agreement were extracted and calculated if the study’s author provided sufficient information. Data on the studies, such as sensitivity, specificity, and the rates of overtriage and undertriage, were calculated and extracted for diagnostic accuracy. If there were any irreconcilable differences in opinion between the first two authors, RB provided the final judgement.

The data that met the study objectives were limited, and several authors were contacted for additional information. Some authors responded with the requested information, while others provided limited yet valuable information. Still, others stated that the requested data was destroyed in accordance with their institutional regulations for retaining data for a maximum of ten years.

### Quality assessment and risk of bias

Because this review focused on two types of studies (interobserver reliability and diagnostic accuracy), two methodological quality assessment tools were used. The consensus-based standards for the selection of health measurement instruments (COSMIN) checklist [23] was used to assess the quality of interobserver reliability studies. This checklist was developed to evaluate studies on the properties of health measurement instruments. This domain contains standards on design requirements and statistical methods in each study. Assessments are classified as very good, adequate, doubtful, or inadequate based on the scores of the items in the interrater reliability domain. The overall score for each study was the lowest score for any of the items in the domain.

To assess the quality of the studies on diagnostic accuracy, the modified Quality Assessment of Diagnostic Studies (QUADAS-2) tool was used [24]. This assessment tool has four domains: patient selection, index test, reference standard, flow, and timing. Each is specifically designed to assess the study’s risk of bias, and the first three evaluate the study’s applicability. To ensure the validity of this tool, a pilot study was performed to test the modified QUADAS-2 before applying it to all included studies.

### Data synthesis

Before conducting this review, a meta-analysis was considered by pooling the values for interobserver reliability (coefficient kappa) and diagnostic accuracy (sensitivity, specificity, PPV, and NPV) separately. Heterogeneity also was planned to be assessed using *I*2 and χ2 tests. However, after extracting the data, there were not enough studies to compare them. There were only three interobserver reliability studies and two with inadequate data for diagnostic accuracy. Unfortunately, the extracted data were similar in only two studies on the objectives of interobserver reliability, while in the studies on diagnostic accuracy, the extracted data were not in the same domain. Thus, conducting a meta-analysis with such data is unreasonable, and narrative analysis was considered after a group discussion by the review team. After tabulating the extracted data, the similarities and variances of the included studies were compared. All relevant studies were grouped based on their aim and objective, either in the interobserver reliability or diagnostic accuracy groups. Then, specific data for each group were extracted. For instance, the following measures of interobserver reliability were extracted: kappa agreement, Spearman, and Pearson. For diagnostic accuracy, these measures were extracted: sensitivity, specificity, PPV, and NPV. If the study did not mention these values, reviewers calculated them manually.

### Study selection

**Fig. 1:**
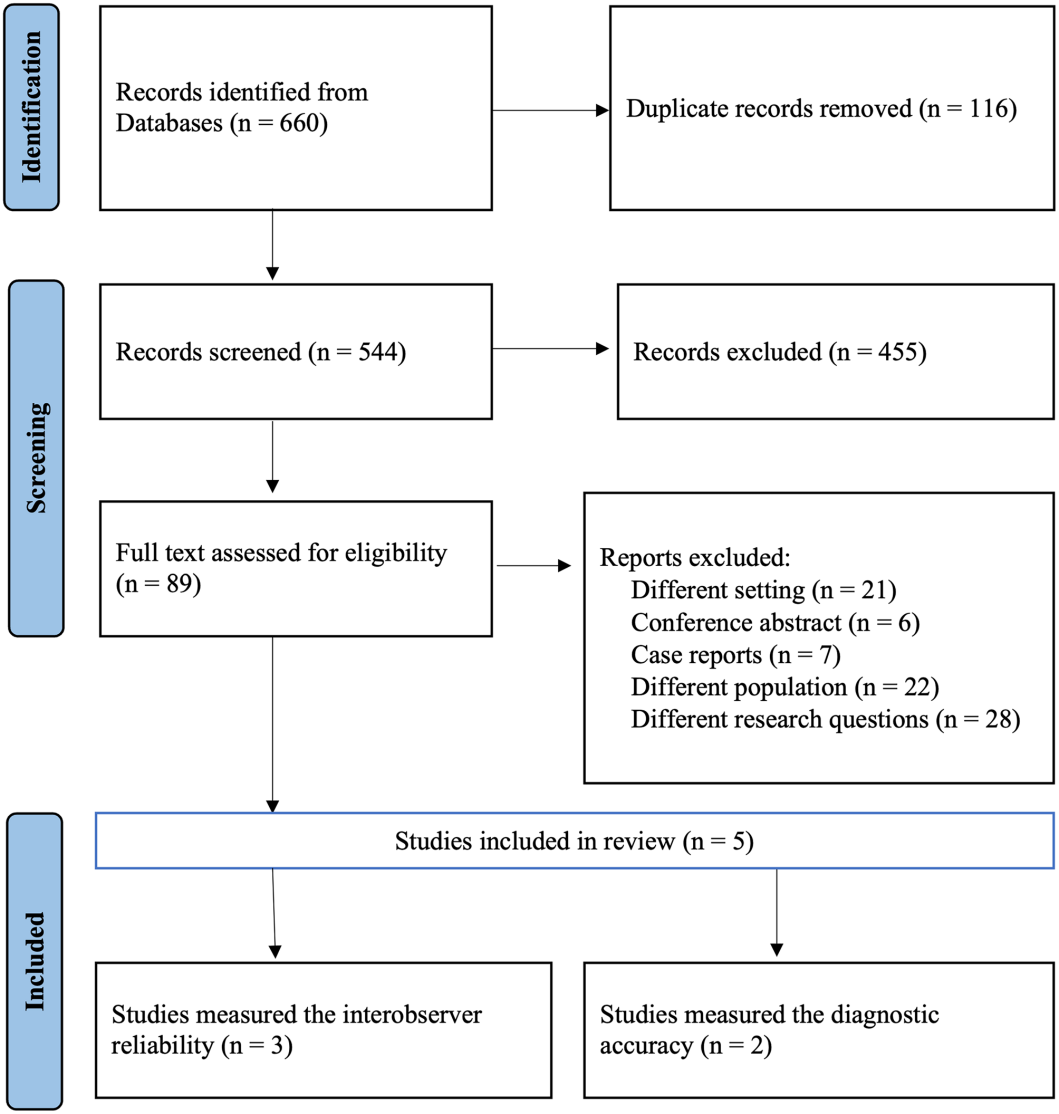
PRISMA diagram

The search strategy, review of grey literature, and manually checking bibliographies yielded 660 papers for both searches. Among these, 116 were duplicates, leaving 544 for titles and abstract screening. After excluding irrelevant papers by title (*N* = 455), irrelevant conference abstract (*N* = 6), adult TBI (*N* = 22), different settings (*N* = 21), and not reporting sufficient information and different research aims (*N* = 28), the review eventually contained four full articles and one abstract with sufficient data. The study selection process for both searches is displayed in the PRISMA flowchart (Fig. 1).

### Study characteristics

All studies were conducted in high-income countries (HIC) with regionalised trauma care: the UK [25, 26], the USA [27, 28], and Spain [29]. All studies were published in the last decade, 2012–2022. Furthermore, data for one study were collected retrospectively and prospectively [26], while four studies were retrospective, with the periods for data ranging from 1 to 22 years. The total number of patients in the included studies was 3404, with a range of 98–1711 participants per study and a median of 499 patients. The interquartile range (IQR) was 141.5–1311. All included studies were conducted on paediatric patients, and only one study excluded children under 5 years of age [28]. Moreover, the studies conducted in the UK included all patients less than 16 years of age and patients less than 15 years of age [26]. However, studies conducted in the USA included patients up to 18 years old [27, 28]. The study conducted in Spain did not specify the ages included. However, the IQR for ages included in the study was 2.49–11.23 [29]. All studies treated all paediatric patients as one group, and one study analysed the patients’ data regarding whether they were younger or older than 3 years of age [27]. Three studies were conducted only in a single level 1 trauma centre and EMS system [27–29], while two studies reviewed patients’ medical records nationally in the UK [25, 26]. The characteristics of the studies are shown in Table 2.

**Table 2:** Characteristics of included studies

	*Publication (year)*	*Study design*	*Country*	*Study period*	*Demographic*	*Sample size*
***Interobserver reliability studies***	Nesiama et al. (2012)	Medical record review	USA	2000–2005	5–18 years	185
	Drews et al. (2019)	Retrospective review	USA	1994–2016	< 18 years	1711
	Nuttall et al. (2018)	Cross-sectional study	UK	September 2009–February 2010	< 15 years	911
***Diagnostic accuracy studies***	Lyttle et al. (2013)	Retrospective clinical registry data	UK	2007–2012	< 16 years	334
	Cabrero Hernández et al. (2022)	Observational cohort study; data collected prospectively and retrospectively	Spain	2002–2017	Not specified	98

### Risk of bias assessment

Two reviewers used the COSMIN domain of the interrater reliability studies to evaluate the included studies. Two studies were classified as inadequate due to a lack of calculated Pearson or Spearman correlations [26, 27], while one study was classified as adequate because it included the calculations of several interobserver reliability tests in addition to kappa: ICC, Pearson, and the Spearman correlation coefficient [28]. The overall assessment scores for the studies of interobserver reliability are shown in Table 3.

**Table 3:** Overall COSMIN score and the reliability measures used

	*Reliability measure*					*COSMIN score*
	*Cohen’s kappa*	*Intraclass correlation coefficient*	*Concordance correlation*	*Pearson correlation coefficient*	*Spearman’s correlation*	
Nesiama et al. (2012)	√	-	√	√	-	Adequate
Drews et al. (2019)	√	-	-	-	-	Inadequate
Nuttall et al. (2018)	√	-	-	-	-	Inadequate

**Fig. 2:**
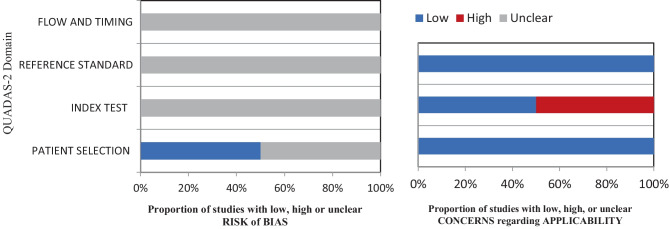
QUADAS-2 domain risk of bias and applicability assessment

The studies exploring diagnostic accuracy were evaluated as having a high, low, or unclear risk of bias. The unclear study was a conference abstract, and the author could share only limited data. The studies’ quality was assessed by two authors independently (SA and NA), with conflicts resolved by discussion or by involving a third author (RB). The risk of bias and the applicability assessment for diagnostic accuracy studies are shown in Fig. 2.

### Results of individual studies

In terms of interobserver reliability, all three studies investigated the level of agreement between paramedics and ED physicians in using GCS as a triage tool [26–28]. Two studies showed moderate agreement ranging from 0.486 to 0.599 [26, 27], while Nesiama et al. (2012) indicated that there was good agreement *k* = 0.69, CI = 0.57–0.81 between paramedics and ED physicians when calculating the GCS for children with TBI. Moreover, Drews et al. (2019) studied the GCS interobserver reliability only for children under three. Their study showed fair agreement with Cohen’s kappa of 0.348, CI = 0.237 to 0.458. Both Drews et al. (2019) and Nuttall et al. (2018) examined the level of agreement with a GCS cut-off of 15. Furthermore, only Drews et al. (2019) used ICD-9 as a reference for diagnosing children with TBI, and they were the only researchers who examined the level of agreement for a GCS sub-score. Other tests of the level of agreement were used to assess interobserver reliability, such as Pearson’s and Spearman’s tests.

The studies of diagnostic accuracy investigated different triage tools. Cabrero Hernández et al. (2022) focused on the factors of paediatric TBI that led to morbidity and mortality in different settings, such as prehospital and the PICU. They extracted GCS accuracy values, such as sensitivity (86.67%) and specificity (71.43%) ([95% CI]: 0.74–0.96). Furthermore, Lyttle et al. (2013) published an abstract in which the diagnostic accuracy of different triage tools, such as PTS and PTT, was tested for children who weighed less than 35 kg with targets for undertriage < 5% and overtriage < 50%. They showed that both tools failed to triage paediatric TBI accurately. The PTS overtriage rate was 10%, and the undertriage rate was 62%, while triage tape had an overtriage rate of 18% and an undertriage rate of 68%. For more illustration, a summary of the studies’ findings is presented in Table 4.

**Table 4:** Summary of the Findings of the included studies

*Interobserver reliability studies*		***Triage tool***	***Reference standard***	***GCS cutoff***	***Sample size***	***Both positive***	***ED negative***	***Scene negative***	***Both negative***	***Cohen’s kappa***	***SE of kappa***	***95% CI***	***Level of agreement***	***Pearson correlation coefficient***	***Concordance correlation***
	Nesiama et al. (2012)	GCS	-	-	185	-	-	-	-	0.69	-	0.57–0.81	Good	0.841	0.839
	Drews et al. (2019)	GCS	ICD-9	15	Age > 3	399	127	232	690	0.486	0.023	0.441–0.531	Moderate	-	-
					1448										
					Age < 3	76	29	57	101	0.348	0.056	0.237–0.458	Fair	-	-
					263										
	Nuttall et al. (2018)	GCS	-	15	911	190	73	78	570	0.599	0.029	0.541–0.656	Moderate	-	-
*Diagnostic accuracy studies*		***Triage tool***	**Reference standard**	***Overtriage rate ***	***Undertriage rate***	***Predictive values***						***Sensitivity***		***Specificity***	***95% CI***
						TP	FN	FP	TN	PPV%	NPV%				
	Lyttle et al. (2013)	PTS	AIS ≥ 3	10%	62%	-	-	-	-	-	-	-		-	-
		Trauma tape		18%	68%	-	-	-	-	-	-	-		-	-
	Cabrero Hernández et al. (2022)	GCS	ICD-9	-	-	19	3	22	54	46.34%	95%	86.67%		71.43%	0.74–0.96

## Discussion

To our knowledge, this systematic review is the first of its kind to investigate the publications of paediatric TBI triage tools, interobserver reliability between paramedics and emergency clinicians, and diagnostic accuracy of triage tools in the prehospital setting to identify children with head injuries. From the results of the review, there are few publications related to diagnosing paediatric TBI in out-of-hospital settings. Three publications focused on the interobserver reliability of GCS and its level of agreement when calculated in the prehospital setting and in-hospital ED [26–28]. Two studies showed a moderate agreement of GCS when calculated for paediatric patients with TBI [26, 27]. Drews et al. (2019) indicated that the level of agreement declined in younger age groups by showing that the level of agreement in children under 3 years old is fair: *k* = 0.348 with 95% CI [0.237, 0.458]. In addition, Nesiama et al. (2012) revealed that the level of agreement is good, with *k* = 0.69 with 95% CI [0.57, 0.81], in patients over 5 years old. These results showed that the level of agreement of the triage tools, specifically GCS, varies depending on one main factor: the age group [28].

Moreover, several studies recommended using components of GCS to identify patients with TBI, especially in time-critical environments like the ED and prehospital [30–32]. In this review, all the included studies evaluated raw GCS scores rather than grouped GCS scores. However, Holmes et al. (2005) conducted a prospective study to compare the GCS with its components in children presenting to EDs with TBI. They showed no significant difference in identifying TBI by GCS or its components. However, of all the GCS sections, the verbal part performed better, followed by eye measure, while the motor part had the worst performance in children.

Only two studies measured the diagnostic accuracy of triage tools. One was a conference abstract, and the author cooperated by providing additional information. Lyttle et al. (2013) measured the overtriage and undertriage rates for children with TBI in a prehospital setting. They examined several international and national UK-based triage tools with target rates of < 5% undertriage and < 50% overtriage. They showed that no existing tool accurately triaged paediatric patients with TBI in prehospital settings. However, PTS performed better than other nationally used triage tools in the UK, with an overtriage rate of 10% and an undertriage rate of 62%.

The diagnostic accuracy of PTT was tested in children who weighed less than 35 kg. Its performance was insufficient, with an overtriage rate of 18% and an undertriage rate of 68%. This study showed that both triage tools had high undertriage rates, so the further study should be conducted to optimise the undertriage rates of paediatric TBI prehospital triage tools. This result contrasted with other studies on children with trauma in general, and it is well-known that paediatric patients are overtriaged by paramedics [25]. Drews et al. (2019) indicated that around 27% of 1711 paediatric patients triaged with lower GCS in the prehospital setting than in the ED, indicating possible overtriage. Although these studies showed the inconsistency of GCS, it is still considered the predominant neurological scale.

Regarding diagnostic accuracy, Cabrero Hernández et al. (2022) explored the prognostic factors for morbidity and mortality in severe TBI. The research team could extract and calculate GCS accuracy predictive values from their data. In that study, GCS showed high sensitivity (86.67%) and high specificity (71.43%). However, it is considered a challenge when calculated for paediatric patients [33–35]. Unfortunately, no other study has explored the diagnostic accuracy of GCS to provide a comparison. Thus, from this review, it should be admitted that the GCS practise shall continue, and further examination of its accuracy is recommended.

## Future research

Due to the limited availability of published studies on the interobserver reliability of paediatric TBI triage tools and their diagnostic accuracy, it is highly recommended that studies be conducted to investigate both aspects to obtain a better understanding of which triage tools are appropriate for use in out-of-hospital settings to identify children with head injuries. It is important to further explore the level of agreement between paramedics and ED experts. Experts should evaluate prehospital care for children with TBI to obtain a valid and reliable triage tool to provide appropriate care, starting with the people who provide initial care to injured children. This can be achieved by conducting further studies to examine the diagnostic accuracy of existing triage tools for children with TBI, specifically in the out-of-hospital field.

## Limitations

This review was reported following the PRISMA guidelines to ensure its validity. However, several limitations must be addressed in this review. For instance, the review team comprehensively searched the grey literature and manually checked the lists of references to confirm that all relevant studies had been included. However, there is no guarantee that no published paper is missing. Furthermore, one criterion of the protocol was to exclude non-English papers, which might have resulted in a gap in the results. It was planned to conduct a meta-analysis in this review. However, a narrative review was preferred due to the limited availability of similar data. There are some additional limitations related to the papers included in the study. For instance, all the included papers were conducted retrospectively, and some had small sample sizes. Also, there were limitations related to the triage tools used. It was not identified in the papers whether the paediatric versions or the adult versions of the triage tools were used. Moreover, in studies measuring the level of agreement of GCS between paramedics and ED doctors, it was difficult to confirm whether the variations were caused by changes in a patient’s condition or miscalculation by the caregiver. Thus, future studies should consider these limitations when designing their approach.

## Conclusion

Paediatric TBI affects children worldwide, and prehospital providers play an essential role in mitigating secondary injuries. This can be done by providing appropriate care to the injured child, which starts with triage and transferring the child to a proper healthcare facility. This review aimed to evaluate the triage tools used in the prehospital setting from different perspectives. One was to measure the level of paediatric TBI triage tools when applied in prehospital and in-hospital settings. The second aim was to explore the diagnostic accuracy of these tools in identifying children with head injuries in out-of-hospital settings. Five papers were included in this review, three related to interobserver reliability. This review showed that the level of GCS agreement between paramedics and ED doctors was low, and the level of accuracy in diagnosing children with TBI in the prehospital field was poor. This may cause further harm to patients through overtriage or undertriage. Thus, further studies are recommended to improve the prehospital management of children with head injuries.

## Data Availability

The data supporting the findings of this study are available upon request. Please contact Sara Alsuwais at Sara.suwais@gmail.com for access to the data used in this research. We are committed to transparency and openness in research and will provide the data to interested parties for legitimate research purposes.
